# Roux stasis syndrome with a diverticulum: lumen-apposing metal stent placement to straighten the lumen and restore the flow

**DOI:** 10.1055/a-2559-9469

**Published:** 2025-03-27

**Authors:** Sid Ahmed Alioua, Jérôme Rivory, Florian Rostain, Pierre Chenet, Alexandru Lupu, Thierry Malaval, Mathieu Pioche

**Affiliations:** 1 660850 Hepato-gastro-enterology Unit, Beni Messous University Hospital Center, Beni Messous, Algeria; 2 Gastroenterology and Endoscopy Unit, Edouard Herriot Hospital, Hospices Civils de Lyon, Lyon, France; 3 Digestive Diseases Department, Argony Clinic in Pringy, Annecy, France

Roux-en-Y esophagojejunostomy is commonly performed after total gastrectomy, especially in oncological and bariatric surgery. However, it is often associated with complications 1 2 , one of which is Roux stasis syndrome, characterized by symptoms of nausea, vomiting, and postprandial bloating due to impaired digestive flow at the anastomotic site or within the Roux limb 3 . These symptoms can have a significant impact on a patientʼs quality of life and require innovative approaches when conventional treatments fail.

This case presents a patient who developed chronic Roux stasis syndrome three years after undergoing a Roux-en-Y esophagojejunostomy. An excessively long blind jejunal limb had created a diverticulum ( Fig. 1 **a** ), which exacerbated her symptoms due to stasis and misdirected food flow into the diverticulum rather than the lumen. Conservative therapies did not provide adequate relief, so endoscopic intervention was considered.

**Fig. 1:**
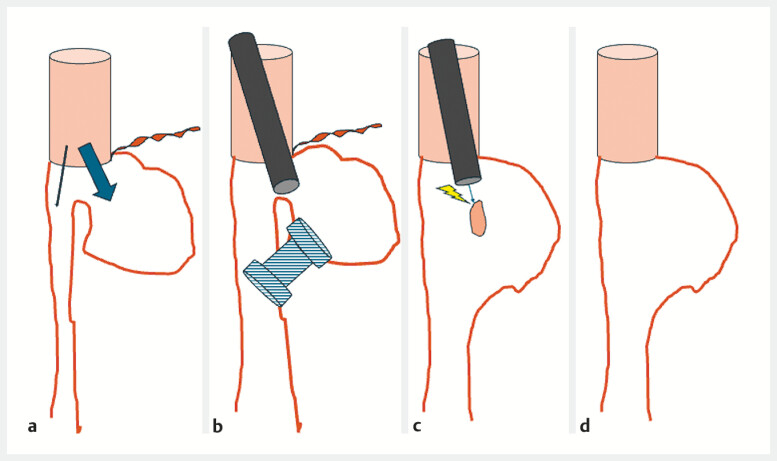
Schematic description of the procedure. **a** Initial aspect with a blind jejuna loop and the main lumen. **b** Placement of a lumen-apposing metal stent between the terminal part of the jejunum and the main lumen. **c** Mucosal bridge section remaining after removal of the stent. **d** Final aspect after section of the spur.

A lumen-apposing metal stent (LAMS) was used to address the anatomical and functional abnormalities causing Roux stasis syndrome. A 20-mm LAMS was used to improve flow by creating communication between the two segments ( Fig. 1 **b** , Video 1 ), resulting in immediate symptomatic relief. The stent will be removed in three months with simultaneous resection of the residual mucosal spur ( Fig. 1 **c** ).

Roux stasis syndrome with a diverticulum: lumen-apposing metal stent placement to straighten the lumen and restore the flow.Video 1

LAMS provides an effective, minimally invasive approach to the treatment of Roux stasis syndrome, offering an alternative to surgery in selected cases. Future studies are needed to validate its long-term efficacy and role in broader management strategies for this syndrome.

Endoscopy_UCTN_Code_TTT_1AS_2AB

## Notes

Endoscopy E-Videos https://eref.thieme.de/e-videos
*E-Videos* is an open access online section of the journal *Endoscopy* , reporting on interesting cases and new techniques in gastroenterological endoscopy. All papers include a high-quality video and are published with a Creative Commons CC-BY license. Endoscopy E-Videos qualify for HINARI discounts and waivers and eligibility is automatically checked during the submission process. We grant 100% waivers to articles whose corresponding authors are based in Group A countries and 50% waivers to those who are based in Group B countries as classified by Research4Life (see: https://www.research4life.org/access/eligibility/ ). This section has its own submission website at https://mc.manuscriptcentral.com/e-videos .
